# Complete mitochondrial genome sequences of five rockfishes (Perciformes: *Sebastes*)

**DOI:** 10.1080/23802359.2018.1495130

**Published:** 2018-08-10

**Authors:** Michael W. Sandel, Andres Aguilar, Vincent P. Buonaccorsi, Jennifer Herstein, Oleg Evgrafov

**Affiliations:** a Department of Biological and Environmental Sciences, University of West Alabama, Livingston, AL, USA;; b Department of Biology, California State University Los Angeles, Los Angeles, CA, USA;; c Department of Biology, Juniata College, Huntingdon, PA, USA;; d Department of Psychiatry and Behavioral Sciences, University of Southern California Medical Center, Los Angeles, CA, USA;; e SUNY Downstate Medical Center, Brooklyn, NY, USA

**Keywords:** Mitochondrial genome, lifespan, *sebastes aleutianus*, *Sebastes minor*, *Sebastes nigrocinctus*, *Sebastes rubrivinctus*, *Sebastes steindachneri*

## Abstract

Rockfishes of the genus *Sebastes* rank among the longest-lived vertebrate animals. In order to facilitate comparative genomic research in animal longevity, the complete mitochondrial genome sequences are presented for *Sebastes aleutianus, Sebastes minor, Sebastes nigrocinctus, Sebastes rubrivinctus*, and *Sebastes steindachneri*.

Within the Perciformes genus *Sebastes* (Rockfishes) rank among the most long-lived bony vertebrates, with age estimates of multiple species exceeding 150 years (Cailliet et al. 2001). Rockfishes have been notable subjects of the comparative biology of aging, and the mitochondrial genome has provided insight into the evolution of lifespan within the genus (Heras et al. 2011, 2015; Hua et al. 2015). The Free Radical Theory of Aging posits the electron transport chain as a fundamental determinant of aging and lifespan, via the production and escape of free radicals from the mitochondrion (Harman 1992). We sequenced the complete mitochondrial genomes of five rockfish species to facilitate comparative functional genomics research relevant to the study of aging. This is the first report of complete mitochondrial sequences for *Sebastes aleutianus* (*n* = 2), *Sebastes nigrocinctus* (*n* = 1), *Sebastes rubrivinctus* (*n* = 2), and *Sebastes minor* (*n* = 1). We also report a new mtDNA sequence of *Sebastes steindachneri* (*n* = 1), corroborating results of Jang, Lee, et al. (2016). Specimens were collected via trawl from the eastern North Pacific Ocean. Specimens were identified and fin-clipped by J.R. Hyde, A.J. Gharrett, and Y. Kai. Morphological vouchers were not retained, but nuclear genome sequences are available for species validation from the Nucleotide database at ncbi.nlm.nih.gov. DNA was extracted using the Qiaxcel DNeasy protocol (Qiagen). Library preparation followed TruSeq protocol for illumina paired-end high-throughput DNA sequencing. Data were generated using an Illumina HiSeq 2000 at the University of Southern California. Paired-end mtDNA sequence reads were selectively assembled to build the complete mitochondrial genome using the iterative mapping approach of MitoBim 1.7 (Hahn et al. 2013). The genome of *Sebastes koreanus* (accession KJ775792.1) was used as a reference sequence. Genome assemblies from each of the paired output files were aligned to each other using the MAFFT online alignment tool (Katoh et al. 2017). Consensus sequences for each sample revealed a standard gene order and GC% content for teleost fishes. Genome annotation was conducted using the online MitoFish MitoAnnotator tool (Iwasaki et al. 2013). The Cytochrome Oxidase 1 gene was used to corroborate species identifications made in the field. For each species, a BLAST search of the CO1 sequence against the NCBI Nucleotide database revealed at least 99% identity to accessions of the same species name. Variability among protein-coding and tRNA genes revealed few insertion-deletions and modest levels of substitutional variation. Conversely, the 3′ region of the mtDNA control region exhibited relatively high levels of sequence divergence and copy number variation, consistent with previous observations of rockfishes (Bentzen et al. 1998; Higuchi and Kato 2002; Zhang et al. 2013). Specifically, a 200–300 bp sequence repeat was detected in the 3′ region of the mtDNA control region. This large repeat sequence varied among species and among individuals within species. The algorithm used for genome assembly did not allow precise quantitation of copy number. Seventeen *Sebastes* mitochondrial genomes were obtained from ncbi.nlm.nih.gov for comparative analysis. A minimum evolution phylogenetic analysis, generated with MEGA7 reveals strong support for sister-taxon relationships for species sequenced herein (Figure 1) (Kumar et al. 2016). In this taxon-limited analysis, *Sebastes aleutianus* was sister to *S. steindachneri*, and this clade was sister to *S. minor*. *S. nigrocinctus* was sister to *Sebastes rubrivinctus*. All other relationships were consistent with results of previous studies (Jang, Kim, Kim 2015; Jang, Kim, Oh, et al. 2015; Jang, Lee, et al. 2016; Jang, Oh, Park, et al. 2016; Jang, Oh, Lee, et al. 2016; Jang, Park, et al. 2016).

**Figure 1.**
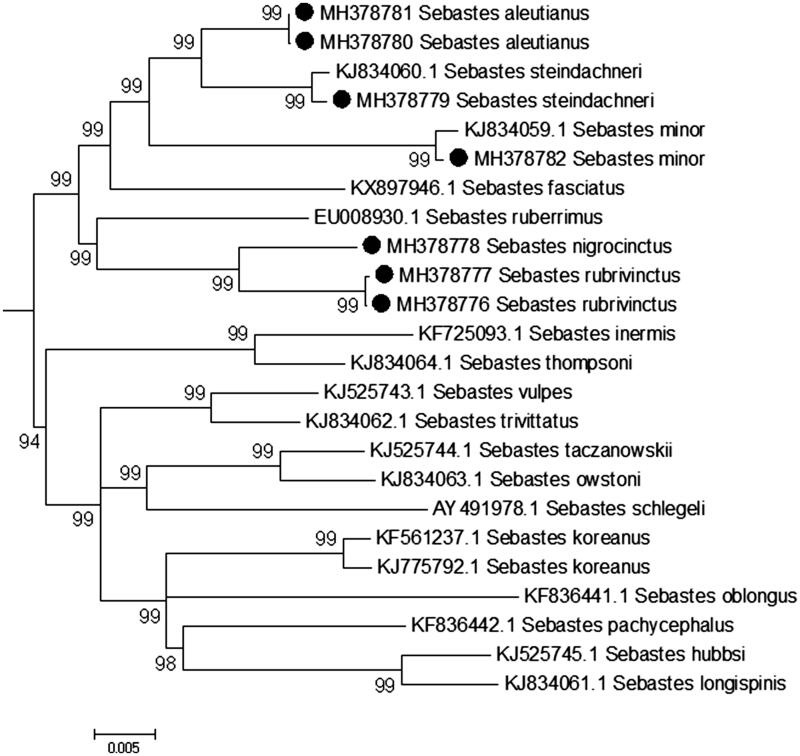
*Sebastes* phylogeny inferred using the Minimum Evolution (ME) method. The optimal tree with the sum of branch length = 1.07547521 is shown. Evolutionary distances were computed using the Maximum Composite Likelihood method and are in the units of the number of base substitutions per site. The ME tree was searched using the Close-Neighbor-Interchange (CNI) algorithm at a search level of 1. All positions containing gaps and missing data were eliminated. There were a total of 13,452 positions in the final dataset. Outgroups are not shown.
